# Metachronous Metastatic Invasive Breast Carcinoma and Metastatic Small Cell Carcinoma Clinically and Radiologically Favoured to Be of Pulmonary Origin: A Case Report

**DOI:** 10.3390/diagnostics16193237

**Published:** 2026-10-07

**Authors:** Adeline-Roxana Bucur, Alexandru Cristian Cindrea, Petra Curescu, Antonia Armega-Anghelescu, Alina Cristina Barb, Flavia Zara

**Affiliations:** 1 Doctoral School, Victor Babes University of Medicine and Pharmacy Timisoara, E. Murgu Square, No. 2, 300041 Timisoara, Romania; adeline.bucur@umft.ro (A.-R.B.); antonia.armega@umft.ro (A.A.-A.); 2 Department of Pathology, Emergency City Hospital, 300254 Timisoara, Romania; flavia.zara@umft.ro; 3 Centre for Artificial Intelligence and Big Data in Emergency Medicine and Clinical Research, Department of Surgery, Victor Babes University of Medicine and Pharmacy Timisoara, 300041 Timisoara, Romania; 4 Department of Oncology, Emergency City Hospital, 300254 Timisoara, Romania; petcurescu@gmail.com; 5 Department of Microscopic Morphology, Victor Babes University of Medicine and Pharmacy Timisoara, E. Murgu Square, No. 2, 300041 Timisoara, Romania; toma.alina@umft.ro; 6 Angiogenesis Research Center, Victor Babes University of Medicine and Pharmacy Timisoara, E. Murgu Square, No. 2, 300041 Timisoara, Romania

**Keywords:** breast cancer, metachronous tumors, small cell carcinoma, therapy

## Abstract

**Background:** In patients with metastatic breast carcinoma, newly identified visceral or pulmonary lesions may represent progression of the known malignancy or, less commonly, a distinct second primary tumor. Histopathological and immunohistochemical evaluation is therefore essential for establishing tumor lineage and guiding treatment. **Case Presentation:** We report the case of a 46-year-old woman diagnosed in 2021 with metastatic hormone receptor-positive/HER2-negative breast carcinoma, presenting with a left breast mass and multiple osteolytic bone metastases. Histopathological examination demonstrated invasive breast carcinoma of no special type, with 98% estrogen receptor expression, 98% progesterone receptor expression, a Ki-67 proliferation index of 15%, and negative HER2 status. She received palliative radiotherapy for painful bone metastases and systemic treatment with letrozole and ribociclib. Five years later, she was evaluated for newly detected liver lesions. Positron emission tomography-computed tomography also demonstrated a metabolically active right centrohilar pulmonary lesion, although a primary lung tumor could not be definitively distinguished radiologically from nodal disease. Liver biopsy revealed a high-grade small-cell neuroendocrine carcinoma with positivity for TTF-1, chromogranin, synaptophysin, and CD56, a Ki-67 proliferation index of 85%, and negative GATA3 expression. These findings, together with the clinical and radiological features, favored a pulmonary origin. The patient was subsequently treated with cisplatin, etoposide, and atezolizumab. **Discussion:** This case illustrates the difficulty of distinguishing progression of metastatic breast carcinoma from a metachronous second malignancy. The markedly different morphology with a discordant neuroendocrine immunophenotypic profile together with the radiological and serological findings, favored a distinct second malignancy rather than progression of the known breast carcinoma. **Conclusions:** New metastatic-appearing lesions in patients with a known malignancy should not automatically be attributed to progression of the original cancer. Re-biopsy and integrated histopathological, immunohistochemical, clinical, and radiological assessment may identify a distinct second malignancy and substantially alter systemic treatment and clinical management.

## 1. Introduction

Breast cancer remains the most prevalent global malignancy and the leading cause of cancer-related mortality [1]. A major clinical challenge is its propensity for distant metastasis to vital organs such as the bone, liver, lung, and brain [2], which represents the principal driver of disease mortality [3]. Although mammary carcinoma can relapse across multiple organ systems, approximately half of advanced cases metastasize to the bone [4].

While historically uncommon, the incidence of multiple primary cancers has increased in recent years, likely driven by advances in diagnostic imaging, prolonged life expectancy, and the adverse effects of interventional modalities [5]. Multiple primary malignancies (MPM) are classified chronologically as synchronous—when the second primary cancer is identified within 6 months of the index tumor—or metachronous—when the secondary lesion is detected beyond this 6-month threshold [6]. Synchronous bilateral breast cancer and metachronous bilateral breast cancer account for up to 4.6% of all new breast cancer cases, respectively [7].

Among cases of non-breast synchronous primaries, malignancies of the female genital organs predominate [8], with germline mutations in the *BRCA1* and *BRCA2* tumor suppressor genes predisposing individuals to both breast and ovarian cancers [9].

Among cases of non-breast metachronous primaries, breast cancer patients face an elevated risk of developing primary lung cancer compared to the general population; this risk is influenced by smoking, radiotherapy, chemotherapy, and estrogen and progesterone receptor status [10]. Regarding metachronous tumors, primary lung malignancies are the most frequent, typically detected within 3 years following breast cancer surgery [11]. Indeed, a previous breast cancer diagnosis is recognized as an independent risk factor for developing lung cancer [12]. Conversely, adjuvant chemotherapy is not significantly associated with an increased risk [13] and has even been correlated with a lowaer subsequent risk of lung cancer [14]. In patients with a history of breast cancer, pulmonary lesions are typically metastatic recurrences from the primary disease. However, albeit rare, as in our case, these lesions can sometimes also be a distinct second primary lung cancer [15].

Small cell lung carcinoma arises in the hilum of smokers, with a poor prognosis, high proliferation rate, short doubling time of tumor mass and pronounced tendency for early metastasis [16].

Second primary cancers more often accounted for death in older survivors and in those with early-stage index breast cancer [17].

We present a very rare case of a metachronous metastatic small cell carcinoma 5 years after the diagnosis of a metastatic breast carcinoma.

## 2. Case Presentation

The current paper presents the case of a 46 year-old woman who presented in the Oncological Surgery Department of the Emergency City Hospital in Timisoara, Romania, in May 2021 for lumbar and anterior thoracic pain, nausea and bloating. The symptoms began two months earlier, and gradually worsened in the last 4–5 days.

The patient did not smoke, and her history showed essential arterial hypertension and fibromatous uterus. Upon clinical examination, pallor and facial and inferior extremities edema were noted.

The vital signs of the patient were noted: heart rate of 87 bpm, blood pressure of 165/110 mmHg, and oxygen saturation of 97%.

The patient’s laboratory tests revealed several abnormal findings, including anemia and high alkaline phosphatase and triglycerides. Elevated inflammatory markers were also observed, such as high erythrocyte sedimentation rate.

**Figure 1:**
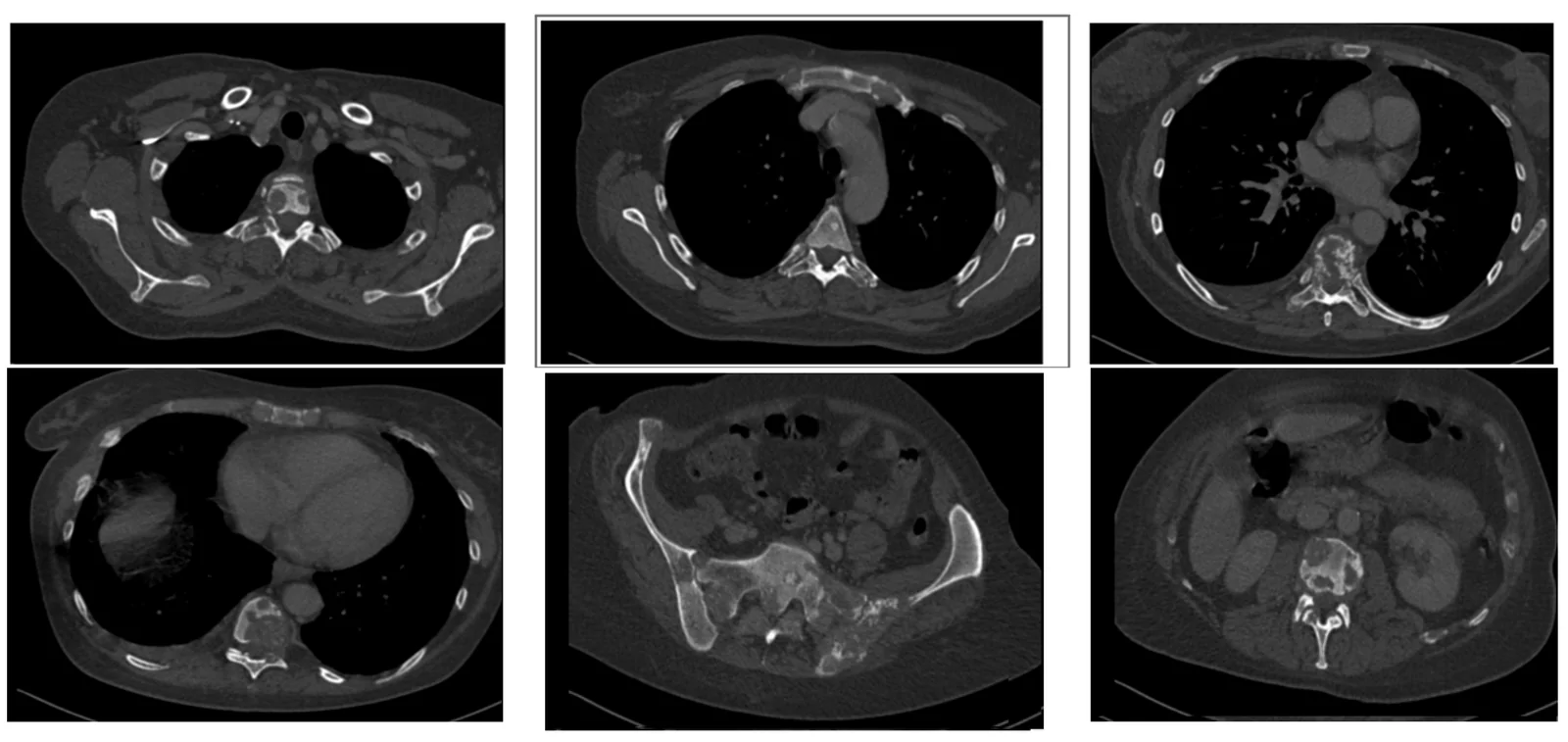
Thoraco-lumbar CT-scan with multiple osteolytic lesions.

Regarding the patient’s thoracic pain, a thoracic angio-computed tomography scan was performed. The computed tomography (CT) scan (Figure 1) revealed multiple osteolytic metastatic lesions in the sternum, vertebrae and ribs, with fractures and cystic lesions in the breasts, bilaterally, the largest one with a diameter of maximum 48 mm in the right breast.

A thoraco-lumbar computed tomography was also performed showing multiple osteolytic metastatic lesions in the thoraco-lumbar vertebrae and pelvic bones.

The abdominal ultrasound was unremarkable.

Regarding the patients nausea and bloating, a gastroscopy was performed showing a hiatal hernia and erosions of the gastric mucosa.

**Figure 2:**
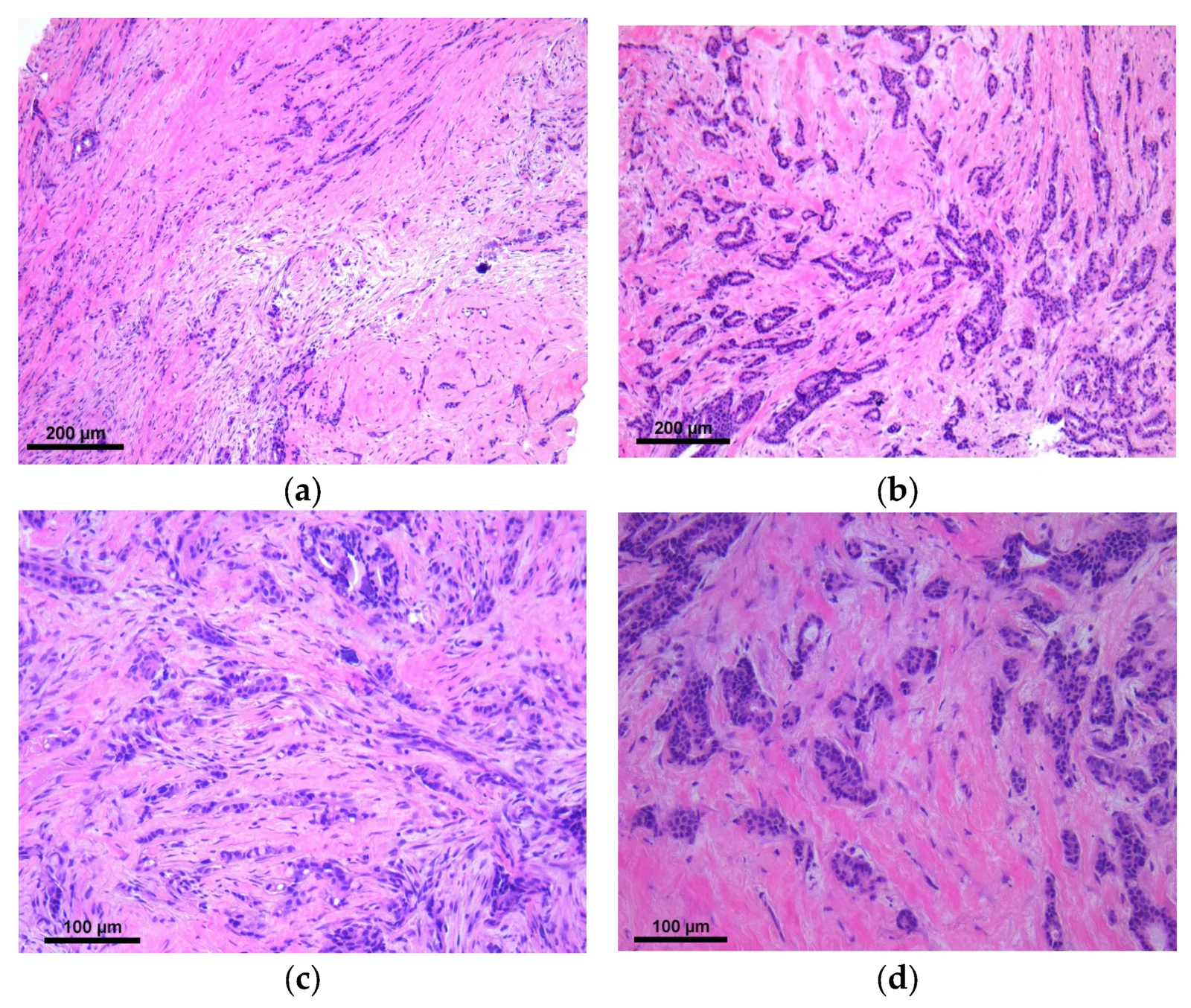
Microscopy: (**a**) Infiltrative trabecular pattern, ob. 10×; (**b**) Infiltrative tubular pattern, ob. 10×; (**c**) Infiltrative trabecular pattern with stromal desmoplasia and microcalcifications, ob. 20×; (**d**) Infiltrative tubular pattern with stromal desmoplasia, ob. 20×.

A surgical exam was performed, detecting a 6 cm tumor in the central quadrant of the left breast, with clinical and ultrasonographical features of malignancy, with no axillary lymphadenopathy. A core needle biopsy was performed and the harvested tissue was fixed in 10% (*v*/*w*) neutral buffered formalin and sent to the Department of Pathology for a histopathological examination (Figure 2).

The macroscopic examination revealed five tissue cylinders of 2 mm diameter and maximum 15 mm length. For diagnostic purposes, serial sections of 4 µm thickness were cut from the paraffin-embedded samples and stained using the standard Hematoxylin–Eosin (HE) method. The microscopical examination showed breast parenchyma replaced by a malignant proliferation of tubules and trabeculae consisting of cells with moderate cito-nuclear atypia. The final diagnosis was breast infiltrating carcinoma NST.

**Figure 3:**
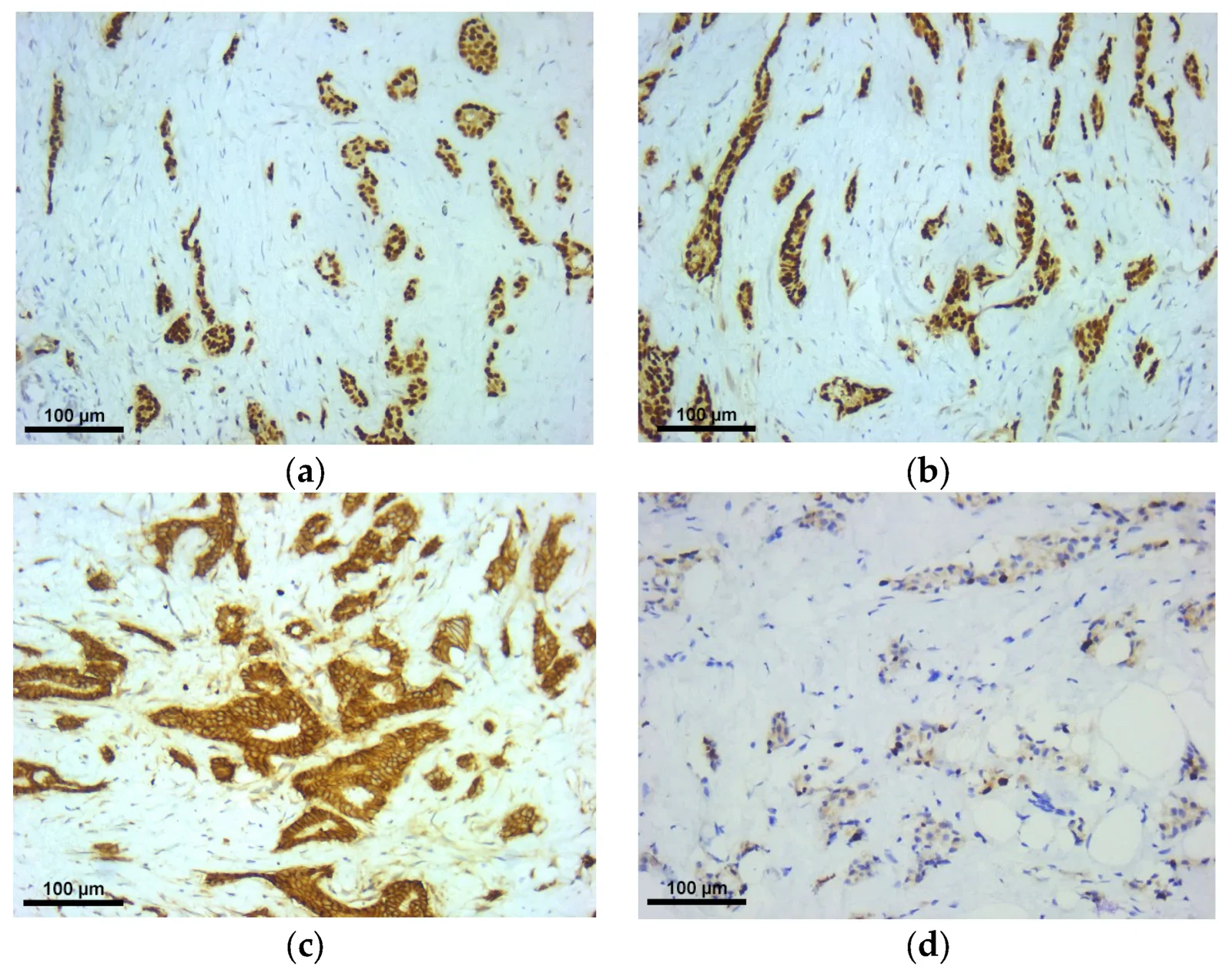
Microscopy: (**a**) Estrogen receptors, ob. 20×. (**b**) Progesterone receptors, ob. 20×. (**c**) HER2 protein, ob. 20×. (**d**) Ki-67, ob. 20×.

Further immunohistochemical (IHC) staining (Figure 3) was performed for therapeutic purposes indicating a luminal A molecular tumor subtype with 98% estrogen receptors (ER), 98% progesterone receptors (PR), a Ki-67 of 15% and a negative human epidermal growth factor receptor 2 (HER2).

The patient underwent antalgic radiation therapy (16 Gy in 4 consecutive sessions), without details about the area involved and a long term treatment for metastatic hormone receptor-positive/HER2-negative breast cancer with Letrozole, Ribociclib and Denosumab.

Five years later, the woman presented for a second time in the Oncological Surgery Department of the Emergency City Hospital in Timisoara, Romania, in May 2026, for the surgical evaluation of liver metastatic lesions.

At the time of this presentation, the patient was receiving systemic therapy for metastatic hormone receptor-positive/HER2-negative breast cancer. The clinical exam showed no abdominal pain, no signs of peritoneal irritation and a normal transit.

**Figure 4:**
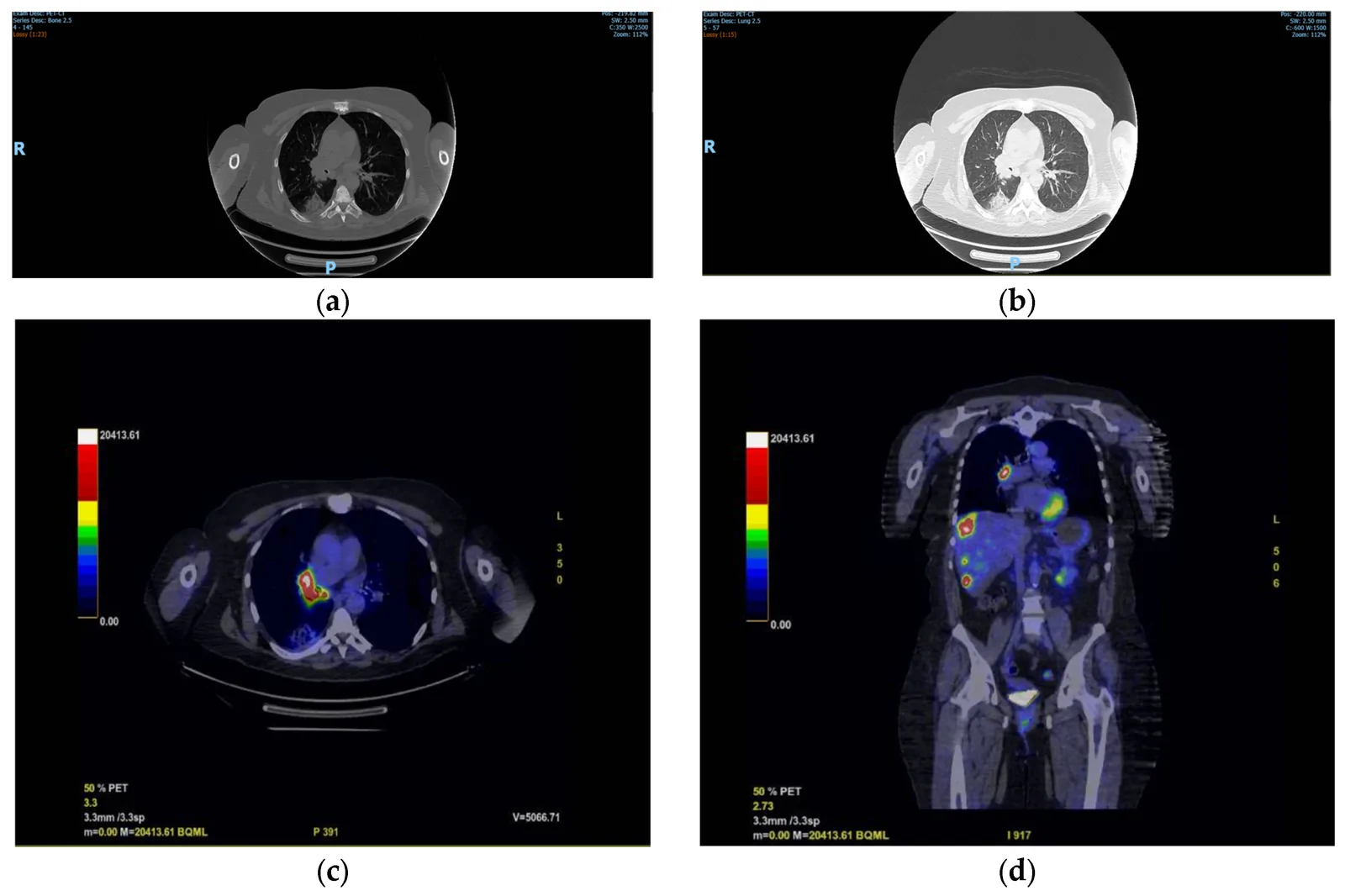
(**a**,**b**) CT-scan with hilar and inferior right lobe lesions, (**c**) axial PET-CT-scan with right pulmonary hilar lesion, (**d**) coronal PET-CT-scan with right pulmonary hilar and liver lesions.

The positron emission tomography-computed tomography (PET-CT) performed prior to the admission (Figure 4) revealed liver metastasis and a hilar lesion in the right lung, metabolically active, most probably a lymph node metastasis, without certainly excluding a primary tumor of the lung. Serum neuron-specific enolase (NSE) was elevated at 60.55 ng/mL.

Given the optimal safety and accessibility of the hepatic lesion, an ultrasound-guided trucut biopsy of the liver was performed and the harvested tissue was fixed in 10% (*v*/*w*) neutral buffered formalin and sent to our Department of Pathology for a histopathological examination. No bronchoscopic biopsy or other pulmonary tissue sampling was performed.

The macroscopical examination described four tissue cylinders with a 2 mm diameter and a maximum length of 7 mm.

**Figure 5:**
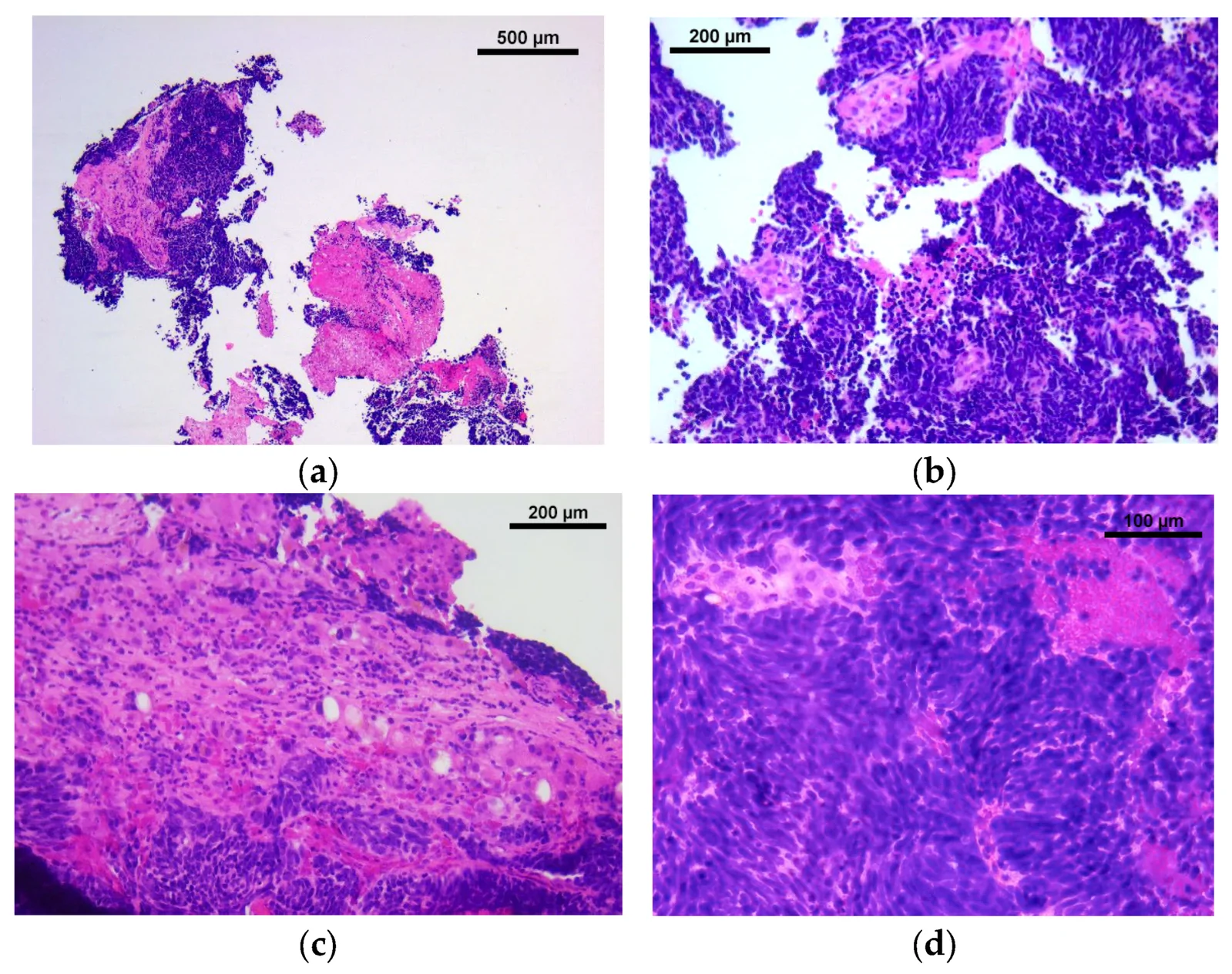
Microscopy: (**a**) Tissue fragments with tumor and necrosis, ob. 4×. (**b**) Tumor with crush artifacts, ob. 10×. (**c**) Tumor infiltrating the hepatic parenchyma, ob. 10×. (**d**) Infiltrating tumor cells with “salt and pepper” nuclei, ob. 20×.

The microscopical examination (Figure 5) described consistent tissue cylinders of liver parenchyma replaced by islands of malignant cells with scant, basophilic cytoplasm and large, hyperchromatic nuclei, with a “salt and pepper” chromatin. Comparing with the initial breast biopsy, the invasive pattern was exclusively solid, with no tubular/pseudoglandular structures favoring adenocarcinoma, and the cells showed typical neuroendocrine features.

**Figure 6:**
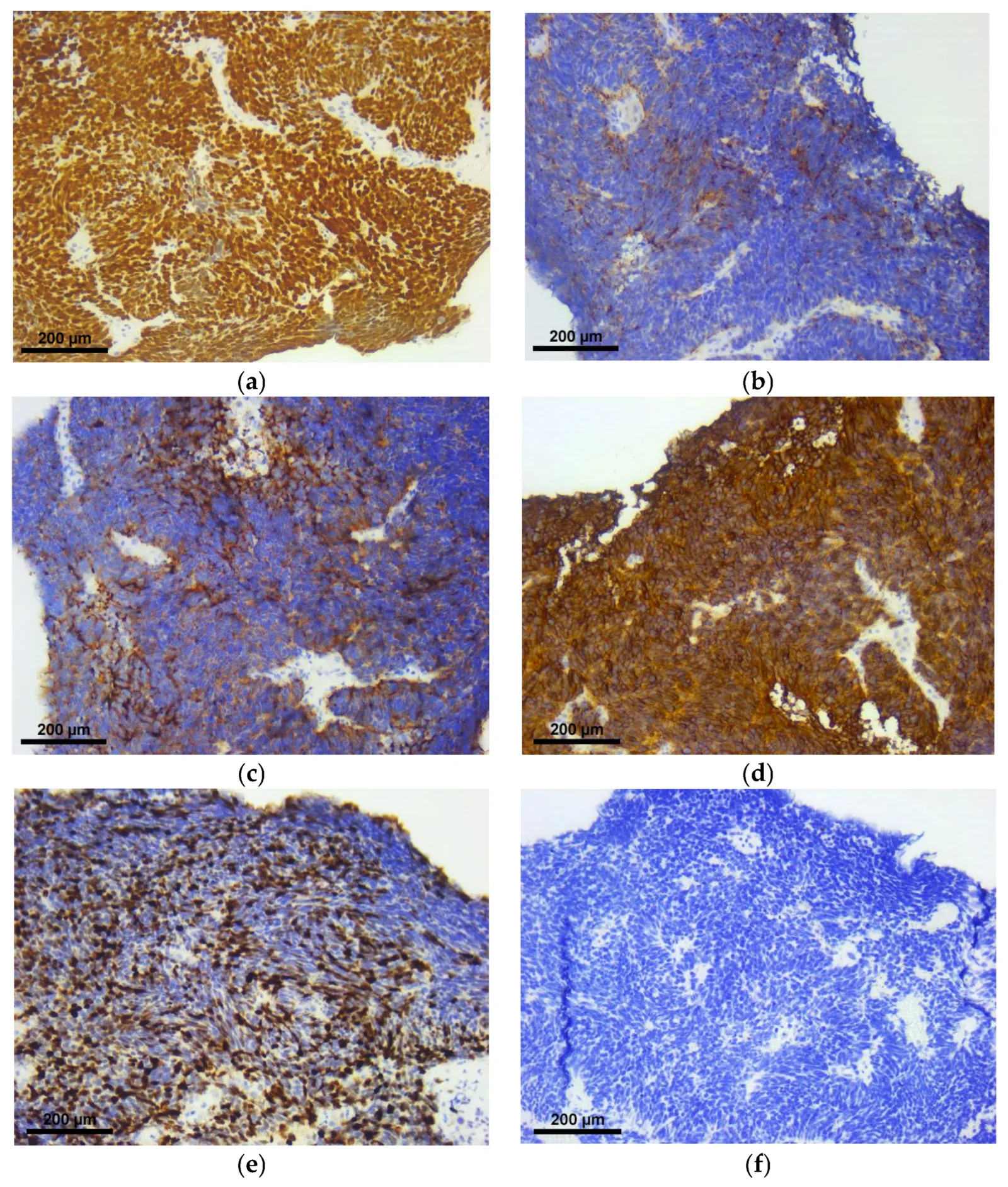
Microscopy: (**a**) TTF-1, ob. 10×. (**b**) Chromogranin, ob. 10×. (**c**) Synaptophysin, ob. 10×. (**d**) CD56, ob. 10×. (**e**) Ki-67, ob 10×. (**f**) GATA3, ob. 10×.

Further immunohistochemical stains were performed for tumor lineage and site of origin (Figure 6). The tumor cells were positive for thyroid transcription factor-1 (TTF-1) and the neuroendocrine markers chromogranin, synaptophysin and CD56, with a Ki-67 proliferation index of 85% and negative for GATA3. Due to the tumor’s distinct neuroendocrine morphology and the inherent limitations of small tissue samples, estrogen receptor (ER), progesterone receptor (PR), and HER2 statuses were not assessed in the hepatic lesion, as they fall outside the standard diagnostic panel for neuroendocrine neoplasms. GATA3 negativity was discordant with the original breast phenotype but was not independently exclusionary. The final diagnosis, based on the correlation with the radiological and serological findings, was liver metastasis of small-cell neuroendocrine carcinoma, clinically/radiologically favored to be of pulmonary origin.

Given the clinically/radiologically favored pulmonary origin and hepatic metastases, the patient was managed as having extensive-stage small-cell carcinoma (Stage IV). Standard frontline management comprises dual-agent chemotherapy—utilizing a platinum compound paired with etoposide—in combination with an immune checkpoint inhibitor. First-line systemic therapy was initiated with cisplatin, etoposide, and atezolizumab. The treatment with Letrozole and Denosumab for the breast cancer continued, but Ribociclib was discontinued due to the risk of neutropenia. The patient is currently under surveillance.

## 3. Discussion

### 3.1. Primary Case Finding & Clinical Dilemma

We present a rare clinical scenario involving a non-smoking female patient diagnosed with a secondary metachronous metastatic small cell carcinoma five years following an initial diagnosis of metastatic breast cancer (MBC) under active oncological surveillance. The incidence of second primary malignancies (SPMs) broadly ranges from 0.99% [18] to 5.8% [19], with metachronous tumors occurring at a 2.4-fold higher frequency than synchronous ones [20]. Although the lung is widely recognized as a predominant site for SPMs [21,22], and prior breast cancer serves as an independent risk factor for lung malignancy [23], distinguishing a new primary pulmonary tumor from metastatic breast disease presents an acute diagnostic challenge. This is particularly true when the lung lesions are small and/or difficult to biopsy or when radiological overlaps—such as irregular margins or central defects—render non-invasive imaging insufficient for definitive differentiation [24,25].

### 3.2. Diagnostic Logic & Immunohistochemical Synthesis

To resolve the diagnostic ambiguity, an integrated approach using computed tomography (CT), whole-body PET/CT, and a targeted tissue biopsy is recommended [26]. In our patient, both imaging studies showed multiple lesions in the right lung and liver. A tru-cut biopsy was performed, the tissue was processed and a stepwise immunohistochemical evaluation followed [26]. The tumor cells were positive for two neuroendocrine markers and had a Ki-67 proliferation index of 85%, confirming a small cell neuroendocrine tumor. In such cases, evaluating TTF-1 (supporting lung origin) and GATA3 (supporting breast origin) serves as a crucial diagnostic tool, alongside expanded combinations like cytokeratin 7, SOX10, and GATA3, which maintain diagnostic utility even in poorly differentiated subtypes [27]. The tumor cells were positive for TTF-1 and negative for GATA3. The final diagnosis, based on the correlation with the radiological and serological findings, was liver metastasis of small-cell neuroendocrine carcinoma, clinically/radiologically favored to be of pulmonary origin.

### 3.3. Critical Limitations & Pathological Mimicry

However, several confounding variables limit absolute certainty, especially on small biopsy samples. First, intratumoral heterogeneity and the lack of absolute sensitivity or specificity in solitary antibody markers constrain definitive interpretation [28]. In this case, ER, PR, and HER2 statuses were not directly evaluated within the liver lesion, preventing a total immunophenotypic concordance with the primary mammary carcinoma. GATA3 negativity alone cannot conclusively exclude a mammary lineage, nor does TTF-1 positivity unequivocally confirm a primary lung origin. This diagnostic overlap is further complicated by the fact that a minority of primary lung carcinomas express ER [29], and metastatic breast clones can dynamically shift their molecular profiles post-therapy [30]. Nevertheless, the combination of a distinct small-cell neuroendocrine morphology, an elevated Ki-67 proliferation index and clinic-radiological correlation strongly favored a secondary primary small cell lung carcinoma (SCLC) over a breast carcinoma metastasis.

### 3.4. Risk Stratification & Epidemiological Context

Epidemiological data underscore the complex prognostic interplay in multiple primaries. Population analyses by Wang et al. using the SEER database indicate that primary breast cancer characteristics—specifically younger age at onset, ductal histology, and ER/PR/TNBC phenotypes—significantly elevate the risk of developing subsequent SPMs. When an secondary SCLC emerges, however, its own tumor stage and age at diagnosis become the dominant determinants of survival. Additionally, age, family history, and HER2 status were independent risk factors for the OS of double primary cancer patients that can help stratify the patients into high or low-risk groups [31].

### 3.5. Therapeutic Approach & Post-Therapeutic Carcinogenesis

Managing concurrent, distinct advanced malignancies requires balancing therapeutic protocols, as primary tumors benefit from therapeutic surgery, whereas metastatic disease is treated with systemic interventions [32]. At the time of her second diagnosis, our patient was undergoing first-line endocrine therapy combined with the CDK4/6 inhibitor ribociclib—a regimen with established survival benefits exceeding 5 years in advanced luminal breast cancer [33]. While high-dose hormonal therapies have demonstrated correlation with long-term solid tumor risks [34], CDK4/6 inhibitors are not significantly associated with secondary carcinogenesis [35]. Although the patient had a history of palliative radiotherapy for bone pain, because the available records do not establish a relevant pulmonary radiation exposure, no causal relationship between radiotherapy and the later small-cell neuroendocrine carcinoma is inferred. The therapeutic regimen was ultimately modified to prioritize the highly aggressive SCLC utilizing cisplatin, etoposide, and atezolizumab [36]. Ribociclib was interrupted during the treatment for the small cell carcinoma, due to the high risk for developing febrile neutropenia, highlighting the challenges of managing dual systemic regimens [37]. Second primary lung malignancies carry some of the lowest 5-year survival rates across all oncological cohorts. Long-term survival with multiple primaries is variable and is influenced by cancer type and stage at diagnosis [38,39].

## 4. Conclusions

This case illustrates the diagnostic challenge of new visceral lesions in a patient with metastatic breast carcinoma, underlining the importance of tissue reassessment and the exclusion of secondary malignancies that dramatically change treatment. The case supports individualized surveillance and, when clinically indicated, genetic counseling according to established criteria rather than universal screening or genetic testing for all patients with multiple primary malignancies.

## Data Availability

The data that support the funding on this study are available from the corresponding author upon reasonable request.

## Abbreviations

The following abbreviations are used in this manuscript:

CT	computed tomography
CDK4/6i	CDK4/6 inhibitors
ER	estrogen receptor
GATA3	GATA protein binding 3
HE	hematoxylin-eosin
HER2	human epidermal growth factor receptor 2
IHC	immunohistochemistry
MBC	metastatic breast cancer
MPM	multiple primary malignancies
NE	neuroendocrine
NSE	neuron-specific enolase
NST	no special type
PET-CT	positron emission tomography-computed tomography
PR	progesterone receptor
RT	radiotherapy
SCLC	small cell lung carcinoma
SEER	surveillance, epidemiology and end result
SPM	second primary malignancies
TNBC	triple negative breast cancer
TTF-1	thyroid transcription factor-1
WHO	World Health Organization
